# Comprehensive Phenological Documentation of *Idesia polycarpa* Using the BBCH Scale: From Bud Development to Dormancy

**DOI:** 10.3390/plants15192942

**Published:** 2026-09-26

**Authors:** Juyan Chen, Mingqi Wang, Chengxi Zhang, Tao Wen, Yanpeng Wang, Yang Xu

**Affiliations:** 1 Key Laboratory of National Forestry and Grassland Administration for Biodiversity Conservation in Southwest Karst Mountains, Guizhou Forestry Science Research Institute, Guiyang 550005, China; 2 School of Ecological Technology and Engineering, Shanghai Institute of Technology, Shanghai 201418, China; 3 Research Institute of Subtropical Forestry, Chinese Academy of Forestry, Hangzhou 311400, China; 4 Guizhou Wetland and Public Welfare Forest Protection Center (Guizhou Yangtze River Upstream Rare and Endemic Fish National Nature Reserve Protection Center), Guiyang 550005, China

**Keywords:** *Idesia polycarpa*, BBCH, phenological stages, woody oil tree, growth cycle

## Abstract

*Idesia polycarpa Maxim (Salicaceae)* is an endemic woody oil tree native to China with multi-purpose economic and ecological applications, including seed oil production, timber utilization, landscape afforestation and biomass exploitation. To date, a unified phenological classification system tailored to this species remains unavailable, restricting standardized cultivation practices and germplasm phenotypic assessment. Here, we developed the first comprehensive Biologische Bundesanstalt, Bundessortenamt und CHemische Industrie (BBCH) scale phenological coding system for *I. polycarpa* through two consecutive full-year phenological monitoring trials in Guizhou Province, Southwest China. We defined eight principal growth stages spanning the complete annual cycle: bud development, leaf expansion, shoot elongation, inflorescence differentiation, blooming, fruit development, fruit ripening, and leaf senescence coupled with dormancy initiation, together with 40 morphologically described secondary stages supported by field photographic documentation. Synchronous vegetative and reproductive growth was observed, while staminate individuals exhibited earlier and longer flowering periods relative to pistillate trees. Three female genotypes were harvested at BBCH principal stage 89 (full fruit maturity) to verify the applicability of the established scale. Distinct genotypic differences in fruit dimensional traits, 1000-seed weight and fatty acid composition were characterized, confirming that BBCH-standardized harvesting eliminates confounding temporal biases for oil quality comparisons. This standardized BBCH coding system offers a universal phenological descriptor for *I. polycarpa* and delivers actionable protocols for orchard fertilization, canopy pruning, pollinizer arrangement and uniform fruit collection. Our findings establish a baseline phenotypic framework to facilitate germplasm discrimination, targeted breeding and intensive sustainable production of this commercially valuable indigenous oil tree.

## 1. Introduction

*Idesia polycarpa* Maxim (Salicaceae, formerly placed in the Flacourticaceae, *I. polycarpa*) is a native woody oil tree species in China. It integrates oil production, timber utilization, ornamental value, and biomass application, embodying significant economic and ecological value [1,2,3,4]. Both seeds and pulp are rich in oil, with the proportion of unsaturated fatty acids reaching 77% in seed oil, among which linoleic acid accounts for up to 62.9% [2]. *I. polycarpa* is a deciduous tree of the *Idesia* genus in Salicaceae, and it is a dioecious tree with some individual plants possessing bisexual flowers [5,6]. Characterized by tall upright stems, straight boles and conspicuous ornamental fruits, the cultivar is widely used in landscaping, ornamental gardening and ecological restoration [7].

Floral and fruit traits act as core phenotypic indicators for cultivar identification and in the taxonomy of woody plants. Mulberry cultivars are classified according to stylar length, fruit color and fruit size [8]. Mulberry presents complex sexual differentiation, including dioecious, monoecious and perfect-flowered individuals [9]. Persimmons produce staminate, pistillate and hermaphrodite flowers, and their cultivar classification is mainly determined by whether fruits can naturally lose astringency [10]. Previous research has divided *I. polycarpa* into seven floral morphotypes based on flower morphological characteristics [6]. Considerable variations in fruit color and fruit size are also observed among different germplasm resources. However, few studies have attempted to classify *I. polycarpa* cultivars relying on floral or fruit phenotypic traits.

The BBCH scale has been widely applied to characterize developmental stages of many economically important tree species, offering practical guidance for field management and selective breeding [8,11]. To fully record the whole life cycle of plants, describe germplasm phenotypic features and guide field cultivation for growers, the extended BBCH system has been successively developed for field crops, weeds and fruit trees [12,13,14]. Accurate coding and morphological description of phenological stages are vital for phenotypic characterization and further research of *I. polycarpa*.

Southwest China, particularly in the Guizhou province, provides highly suitable growing environments for *I. polycarpa*. Local climatic conditions, soil properties and altitude are key factors affecting lipid biosynthesis and oil quality of this species. Current studies on *I. polycarpa* primarily focus on oil extraction technologies and high-efficiency cultivation regimes, while research regarding its phenological characteristics remains insufficient. In the present study, we selected five pistillate and three staminate individuals to establish a complete BBCH coding system for their phenological stages. The primarily vegetative and reproductive phenophases of the six tested cultivars (three female and three male trees) were characterized, covering the full life cycle of different sexual types. This established system can serve as a reliable tool for fundamental research and intensive orchard management of *I. polycarpa*.

## 2. Results

### 2.1. The Phenological Growth Stages of I. polycarpa

Combined with field surveys and BBCH standard specifications, the phenological cycle of *I. polycarpa* was divided into eight major phases. The principal developmental stages 2 (lateral shoot formation and tillering) and 4 (development of harvestable vegetative organs, vegetative propagules and booting structures), which commonly occur in most fruit tree species, were not incorporated into the phenological scale of *I. polycarpa*. Stage 2 was excluded since this scale only characterizes the growth dynamics of the primary central stem. Stage 4 was omitted because its developmental characteristics do not correspond to the growth traits of woody plant species.

Among them, stage 0 (bud development), 1 (leaf development), 3 (shoot development) and 9 (senescence and dormancy) belong to vegetative phases; stage 5 (inflorescence emergence), 6 (flowering), 7 (fruit growth) and 8 (fruit maturity) belong to reproductive phases [15] (Figure 1). We took field photographs to record vegetative and reproductive organs at different phenological stages, and matched the data with monthly maximum, minimum and average temperatures across the whole year (Figure 1).

### 2.2. PGS 0: Bud Development in I. polycarpa

In Guiyang, buds remained dormant until 18 March when the average minimum temperature was 4 °C, and bud burst occurred from 20 March to late March (Figure 1 and Figure 2). Buds germinated rapidly within three days in early April.

00: Buds stayed in a state of deep dormancy, tightly wrapped by intact bud scales. No prominent signs of bud burst were observed before 1 April (Figure 2).

01: Buds began to swell and elongate, and the outer bud scales began to crack slightly. This phase lasted for approximately 10 days beginning on 10 March.

03: Bud swelling was fully completed. Bud scales split obviously, and the green inner scale tips were visible, beginning on 10 March (Figure 2).

04: Deliberately omitted because its developmental characteristics do not correspond to the growth traits of woody plant species.

05: Partial abscission of the outer brown bud scales occurred, and green young buds gradually emerged.

06: Deliberately omitted.

07: All the brown outer scales completely detached from bud tissues. Green buds were fully exposed, while leaf primordia remained unexpanded (Figure 2).

09: Within this bud-development phase, further elongation of buds stopped, and buds attained their maximum length (Figure 2).

### 2.3. PGS 1: Leaf Development of I. polycarpa

The whole process from the separation of the first leaf to full leaf expansion lasted approximately forty days (Figure 1 and Figure 2). The leaf-expansion period serves as a critical indicator for assessing canopy recovery.

10: The first leaf became distinguishable with a complete outline, while individual leaflets were still connected to one another (Figure 1).

11: The first true leaf fully unfolded with a tender green color, and the petiole kept elongating continuously.

12–14: Deliberately omitted.

15: Around 50% of total leaves unfold completely. The leaflet area of the first leaf accounts for 50% of its mature size. Leaflets are separated from petioles with a light green appearance, and lignification proceeds slowly in both leaflets and petioles to form solid tissue.

16–18: Deliberately omitted.

19: Leaves continued to expand until all the foliage fully unfolded and attained their ultimate size. Petioles grew to their final length; leaflets deepened to dark green and increased in thickness. (Figure 2).

### 2.4. PGS 3: Shoot Development of I. polycarpa

The shoot developmental cycle spanned approximately two months, commencing on 20 March and concluding in late April. Each terminal bud generally sprouted only one lateral shoot.

20–29: Deliberately omitted.

30: Shoots initiated elongation, and the central shoot axis started rapid development.

31: Shoots attained 10% of their ultimate length, while leaves remained tightly adpressed to the shoot axis (Figure 2).

33: The length of shoots accounted for 30% of the maximum size. Light green pigmentation emerged on new shoots, alongside tissue thickening (Figure 2).

35: The length of shoots accounted for 50% of the final length, and petioles still remained close to the shoot surface.

39: Shoots grew to more than 90% of their ultimate length. Shoot tissues deepened to dark green, completed lignification and fully matured. Terminal and lateral buds could be distinctly seen (Figure 2).

### 2.5. PGS 5: Inflorescence Development of I. polycarpa

Inflorescences emerged and developed synchronously with leaf expansion during/after the shoot elongation phase. Inflorescences emerged at the same time that leaf expansion reached approximately 50%. The differentiation of staminate inflorescences was first observed on 20 March and lasted about 1.5 months. The differentiation of pistillate inflorescences was first observed on 25 March, and the whole inflorescence developmental process lasted nearly one month until late April. As a typical dioecious tree, *I. polycarpa* produces three types of inflorescences: pure staminate inflorescences, pure pistillate inflorescences, and mixed inflorescences bearing both staminate and pistillate flowers.

40–49: Deliberately omitted.

50: Reproductive terminal buds started to swell (lateral floral buds swell laterally).

51: Inflorescence axes began to elongate and achieved 10% of the final length, and the apex of floral buds showed light green.

52: Inflorescence axes initiated elongation and attained 20% of their final length. Single florets were densely arranged on the axis (Figure 3).

54: Inflorescence axes grew to 40% of their ultimate length, and neighboring florets began to diverge marginally.

56: Inflorescence axes extended to 60% of their final length. Florets acquired a yellow tone, and pedicels finished stretching to a steady size (Figure 3).

57–58: Deliberately omitted.

59: Inflorescence axes reached 90% of the final length. Florets continue to swell with nearly uniform size, and the outer calyx lobes begin to split apart.

### 2.6. PGS 6: Flower Development of I. polycarpa

Staminate flowers entered anthesis five days earlier than pistillate flowers, and declined five days later than pistillate flowers. The flowering duration of pistillate flowers was about 15 days (20 April to 5 May), while staminate flowers bloomed for 25 days (15 April to 10 May). For monoecious individuals, the flowering period of pistillate flowers was consistent with that of pure pistillate trees, and staminate flowers on monoecious trees shared the same flowering period with pure staminate trees.

61: Initial anthesis stage: 10% of total flowers opened. On 15 April, 10% of staminate flowers bloomed, while 10% of pistillate flowers entered anthesis on 20 April (Figure 3).

62: Deliberately omitted.

63: Early full-bloom stage: 30% of total flowers opened. At this stage, the ovaries of pistillate flowers are faintly visible and light green in color (Figure 3).

64: Deliberately omitted.

65: Full-bloom stage: More than half of the flowers fully expanded. The calyxes turn obviously yellow and start to shed. The calyxes of pistillate flowers begin to shed two days earlier than those of staminate flowers (Figure 3).

66: Deliberately omitted.

67: Flower withering stage: All the petals and calyxes of pistillate and staminate flowers abscise, leaving only the inflorescence axes for staminate flowers (Figure 3).

### 2.7. PGS 7: Fruit Development of I. polycarpa

The fruit developmental phase spanned 30 April through 5 May. Fruit development proceeds concurrently with flowering. When over 50% of flowers were fully open, the ovaries had already initiated development even though a small number of petals and calyxes remained.

70: Obvious ovary enlargement could be detected.

71: Fruits reached 10% of the final size. Ovaries started rapid expansion, and petals began to abscise. Physiological fruit drop was rarely observed at this stage (Figure 4).

72: Fruits attained 20% of the final size. Seeds were embedded firmly in the pulp, and both seeds and pulp appeared white (Figure 4).

73: Fruits grew to 30% of the final size and entered the rapid expansion stage with continuous volume increase (Figure 4).

75: Fruits reached 50% of the final size. Fruits presented diverse shapes, including round, elliptical and cordate forms. Bracts at the base of fruit pedicels gradually shed. Pulp turned light yellow and seed coat became gray. Meanwhile, the pericarp thickened and lignified, and fruit size tended to be uniform among individuals. The pericarp surface became glossy (Figure 4).

77: Fruits extended to 70% of their final dimension. Fruits kept developing with a smooth and bright peel in green, red, yellow or orange. Pulp turned light yellow (Figure 4).

78: Deliberately omitted.

79: Fruits extended to 90% of their final dimension, and the pulp remained yellow (Figure 4).

### 2.8. PGS 8: Maturity of Fruit and Seed of I. polycarpa

Fruit and seed maturation occurred from 10 to 31 October. When the peel of *I. polycarpa* fruit turns deep red, orange or yellow, it serves as one of the important indicators of fruit ripening (Figure 5). Most fruits of the tested *I. polycarpa* germplasms exhibited dark red pericarp. The pericarp appeared light red at the color transition stage and turned darker red at full ripeness. A small number of fruits possessed orange or yellow pericarp, and these colorations were intensified with fruit maturation.

81: Fruits initiated ripening and became plump. The pericarp formed a glossy surface.

82–84: Deliberately omitted.

85: Pericarp displayed stable and diverse colors including red, orange and yellow, and no further color change was observed.

86–88: Deliberately omitted.

89: Fruits completed vegetative expansion and entered the full ripening stage, with mature seeds. The flesh becomes pale yellow. Fruits of *I. polycarpa* belong to the indehiscent type, which remain intact without dehiscence after maturation (Figure 4 and Figure 5).

### 2.9. PGS 9: Senescence and Beginning of Dormancy of I. polycarpa

The whole process from initial leaf chlorosis to complete defoliation lasted nearly two months, ranging from 25 October to late December. During this period, all the leaves of male plants shed completely. By contrast, fruits of female plants remain attached to the branches and can persist on the trees until March to April of the next year. With the decrease in temperature, the fruits of female plants turn deeper red, while their luster fades gradually.

91: Shoots terminated growth, and all the leaves remained green.

92: Leaves started to lose chlorophyll and turn yellow, with green and yellow foliage coexisting on branches.

93: Chlorotic leaves began to fall off.

95: Approximately 50% of total leaves abscised.

96: Deliberately omitted.

97: All the leaves shed completely, and trees officially entered the dormancy period (Figure 6).

### 2.10. Validation of Fruit Traits and Fatty Acid Profiles Based on BBCH Stage 89

Based on the BBCH phenological coding system, fruits from three female trees of *I. polycarpa* (Fm-5, Fm-14 and Fm-15) were selected for validation. The codes and corresponding developmental durations of vegetative growth stages of the three germplasm resources were largely consistent (Figure 2). Fruit harvesting was conducted at BBCH principal stage 89 (full fruit ripening), with respective ripening dates of 25 September, 5 October and 20 October. At this stage, fruits of all three germplasm resources were fully ripe, featuring glossy deep-red peel surfaces (Figure 4, Figure 5 and Figure 7A).

All the fruits of the three germplasms were harvested at the identical BBCH phenological stage rather than on a fixed calendar date, which avoids the confounding temporal bias caused by asynchronous maturation among genotypes and enables reliable comparisons of fruit traits and oil quality. Significant differences were detected in fruit longitudinal diameter, transverse diameter, single fruit weight and 1000-seed weight among the three germplasm resources of *I. polycarpa*. Fm-15 exhibited the maximum values for all four morphological traits, Fm-5 the minimum, and Fm-14 an intermediate performance (Figure 7B–E).

Detecting the fatty acid content revealed that the late-maturing, large-fruited germplasm resource of Fm-15 possessed the highest total fatty acid content, followed by the early-maturing, small-fruited Fm-5, while the mid-maturing, medium-fruited Fm-14 had the lowest fatty acid content (Figure 7 F). Distinctions in fatty acid composition were also observed across the three germplasm resources. Fm-5 showed the highest relative content of C16:0, whereas Fm-15 contained the lowest relative content of this saturated fatty acid (Figure 7 G). The relative contents of C16:1, C16:2, C18:1 and C18:2 of Fm-5 and Fm-15 were significantly higher than those in Fm-14 (Figure 7H–K). No obvious disparities were detected for the relative content of C18:3 across all three genotypes (Figure 7L).

## 3. Discussion

In this study, we established the BBCH coding system and described eight principal developmental phases of *I. polycarpa*, covering bud dynamics, leaf development, shoot growth, inflorescence formation, anthesis, fruit development, foliage senescence and dormancy transition, which is similar to the description of *Xanthoceras sorbifolium* by Jiang [14]. In total, 40 secondary phenological stages were elaborated at length, while the chronological progression of key growth phases was displayed in schematic figures.

The plant materials of *I. polycarpa* in this study were collected from Sichuan, Shaanxi and Guizhou Provinces. Among them, Sichuan and Guizhou have relatively similar climatic conditions. Shaanxi marks the northernmost wild distribution limit of *I. polycarpa.* All three locations fall within the natural distribution range of this species, with climate, soil and altitude conditions suitable for its growth. Although seedling individuals differed in flowering time, fruit size and even fruit color, the differences in the timing of various growth and developmental stages were less than ten days. We have described the timings of vegetative growth and fruit development as time intervals rather than discrete time points to rationalize the BBCH scale description.

Similarly to the BBCH descriptions for persimmon, *Xanthoceras sorbifolium*, sand pear and *Sapindus mukorossi* [14,15,16,17], *I. polycarpa* adopts a two-digit BBCH coding system. By contrast, three-digit codes are applied to distinguish principal and secondary stages for litchi [18], mangosteen and longan [19]. In Southwest China, the vegetative growth of *I. polycarpa* consists of three major phenophases: bud development (PGS 0), leaf expansion (PSG 1) and shoot elongation (PGS 3) (Figure 1, Figure 2 and Figure 3). Its vegetative and reproductive growth proceed simultaneously, which is consistent with the growth rhythm of *S. mukorossi* and *X. sorbifolium* [14,16], whereas litchi and longan show asynchronous vegetative and reproductive development which is described by Wei [18] and Pham [20].

Similarly to *X. sorbifolium*, longan, litchi and *S. mukorossi*, they produce far more staminate flowers than pistillate flowers, and the two flower types have staggered anthesis periods [14,16,17,18,20]. Nevertheless, most *I. polycarpa* individuals are dioecious (Figure 3). The staminate flowers on purely male plants bloom earlier and last longer than the pistillate flowers on monoecious trees (Figure 3) [6,21]. However, Xinjiang walnut shows a different pattern of flowering sequence, with its female flowers blooming earlier than male flowers [22]. In recent years, monoecious individuals have been discovered in field surveys, which bear both staminate and pistillate flowers on a single inflorescence [6]. In addition, pistillate flowers of *I. polycarpa* can be divided into at least seven morphotypes based on pistil/stamen length and petal morphology [6]. In practical production, the optimal female-to-male plant ratio for high pollination efficiency is 8:1. Compared with *X. sorbifolium* [23], severe fruit drop caused by low pollination rate due to an unbalanced floral sex ratio rarely occurs in *I. polycarpa*, and the pollination success rate and final yield are not limited according to our previous study. During fruit developmental stage 79, abscission occurs in heavy jujube fruits, whereas this phenomenon does not occur in *I. polycarpa* [19].

Fruit yield, external fruit traits, nutritional profiles, and oil fatty acid profiles are strongly modulated by fruit load, fruit dimension, and peel pigmentation [6,24,25]. Previous work also confirmed that fruit size exerts an impact on the fatty acid concentration and constituent proportions of *I. polycarpa* fruit [6]. Apart from morphological disparities, ripening timing is another critical determinant shaping fruit quality across diverse *I. polycarpa* germplasms. In this context, establishing a standardized BBCH phenological classification system for *I. polycarpa* is essential to unify harvest timing and to eliminate temporal confounding effects during fruit quality evaluation. While continuous dynamic monitoring of fatty acid accumulation across developmental phases was not performed for the three tested genotypes (Fm-5, Fm-14 and Fm-15), all the experimental fruits in the present study were uniformly collected at BBCH principal stage 89 corresponding to full physiological ripeness based on our newly developed BBCH scale (Figure 4 and Figure 5). Subsequent phenotypic and lipid profiling demonstrated that germplasm resources differences in fruit dimensions (large, medium and small fruits; Figure 7A) and natural ripening dates are primary contributors to divergent total oil yields and fatty acid component ratios (Figure 7F–L).

The established BBCH phenological system can provide scientific guidance for daily orchard management of *I. polycarpa*, including rational fertilization, canopy pruning and yield improvement. Topdressing with quick-acting fertilizers during stages 30–59 in the spring can replenish nutrients consumed by blooming and supplementing with phosphorus and potassium fertilizers promotes fruit development during stages 66–79. After fruit harvest and before dormancy, application of slow-release fertilizers in late autumn or early winter is recommended to supply nutrients for floral bud differentiation in the next growing year. Considering the low physiological fruit drop rate and high fruit setting rate of *I. polycarpa,* as well as the divergent flowering and fruiting time among different germplasms, fertilizer management should also take local temperature, humidity and soil nutrient status into comprehensive consideration. Research on phenological characteristics of staminate trees is vital for pollination regulation, fruit setting and final yield formation of *I. polycarpa*. The selection of excellent staminate cultivars must match local climate and altitude conditions. Priority should be given to male individuals with synchronized flowering period, long anthesis duration and high pollen viability to guarantee stable fruit yield.

## 4. Material and Methods

### 4.1. Plant Material

A total of ten five-year-old fruiting *I. polycarpa* trees originating from Sichuan, Shaanxi and Guizhou Provinces of China were selected as plant materials for phenological observation. Among all the individuals, accessions 1, 3 and 4 only bore staminate flowers, while accessions 2, 5, 6, 7, 8, 9 and 10 produced purely pistillate flowers. All the experimental trees were planted in the Germplasm Resource Nursery of Guizhou Forestry Science Research Institute, Guiyang, China (Latitude: 26°44′ N, Longitude: 106°27′ E). Guiyang has a humid subtropical climate. According to meteorological data from the past 30 years, the annual average temperature is 15.3 °C, with a mean maximum temperature of 27.6 °C in July and a mean minimum temperature of 1.6 °C in January. The annual average precipitation is 1129.5 mm.

Field observations on vegetative and reproductive phenophases were carried out throughout the annual growing seasons from 2024 to 2025. Observation intervals were established as occurring every two days or a week, depending on the developmental velocity of each phenological stage.

This study builds on our previous investigations of *I. polycarpa* shoot, flower, inflorescence and fruit, whose characterized developmental stages underpin the present research [6]. The classification criteria for fruit developmental stages, which were defined according to relative fruit size ratios, were quantitatively determined based on longitudinal and transverse fruit diameters [6]. To quantify shoot elongation dynamics across developmental stages, 20 branches were selected from each of the four orientations of ten Idesia polycarpa trees, and their lengths were measured periodically, which allowed the proportional length of shoots at each stage to be determined relative to their maximum seasonal extension.

### 4.2. Determination of Fatty Acid and Composition Content

The maturity stage of fruits was picked from Fm-5, Fm-14 and Fm-15 trees at the principal growth stage 8. Fruits with uniform size, ripeness, and no obvious damage were taken from four directions of each tree with triple repetition for further analysis [6]. Fruit samples were prepared as previously outlined by Zhang [26]. The fatty acid composition was determined according to the methods described by AOCS Am 2-93 [27], and fatty acid profiles were quantified following the protocols of Shi and Zhang [26,27,28].

### 4.3. Data Analysis

The oil content and other parameters of fruit samples were measured in triplicate, and the results were shown as mean ± standard deviation (SD). SPSS 21.0 Statistical software (IBM SPSS Inc., Armonk, NY, USA, accessed on 10 May 2023), data were compared using Duncan’s multiple range test at *p* < 0.05. Software of Origin 2021 (Origin Lab Inc., Northampton, MA, USA, accessed on 10 May 2023) and Adobe Illustrator CC 2018 (Adobe Systems Inc., San Jose, CA, USA, accessed on 13 May 2023) were used for the plot drawing.

## 5. Conclusions

This study established the first standardized BBCH-based phenological grading criteria for *I. polycarpa*. The formulated coding system greatly improves the accuracy of intensive cultivation practices, including irrigation scheduling, fertilization regime, pruning technique, harvest arrangement and integrated pest management. Moreover, this work provides references for predicting anthesis of pistillate and staminate flowers and configuring pollinator trees, thus laying a scientific foundation for improving pollination efficiency and fruit yield. Furthermore, the phenological data acquired in this research can supply key phenotypic evidence to accelerate breeding programs and maximize the ecological and economic benefits of *I. polycarpa*.

## Figures and Tables

**Figure 1:**
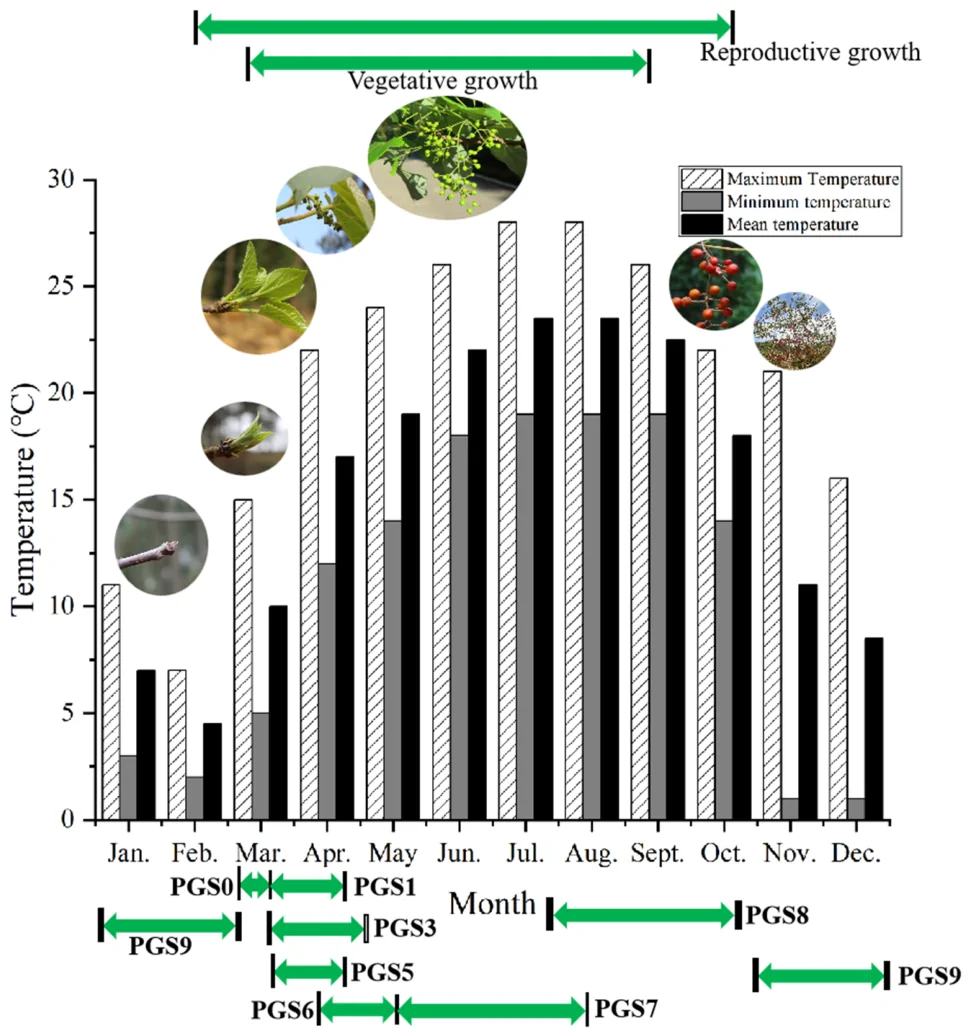
Progression of *I. polycarpa* principal growth stages (PGS) and monthly temperatures following the BBCH scale in Guiyang, Guizhou. Vertical bars represent mean, maximum and minimum monthly temperatures across the experimental period (2024–2025). Horizontal bars stand for the duration of each stage.

**Figure 2:**
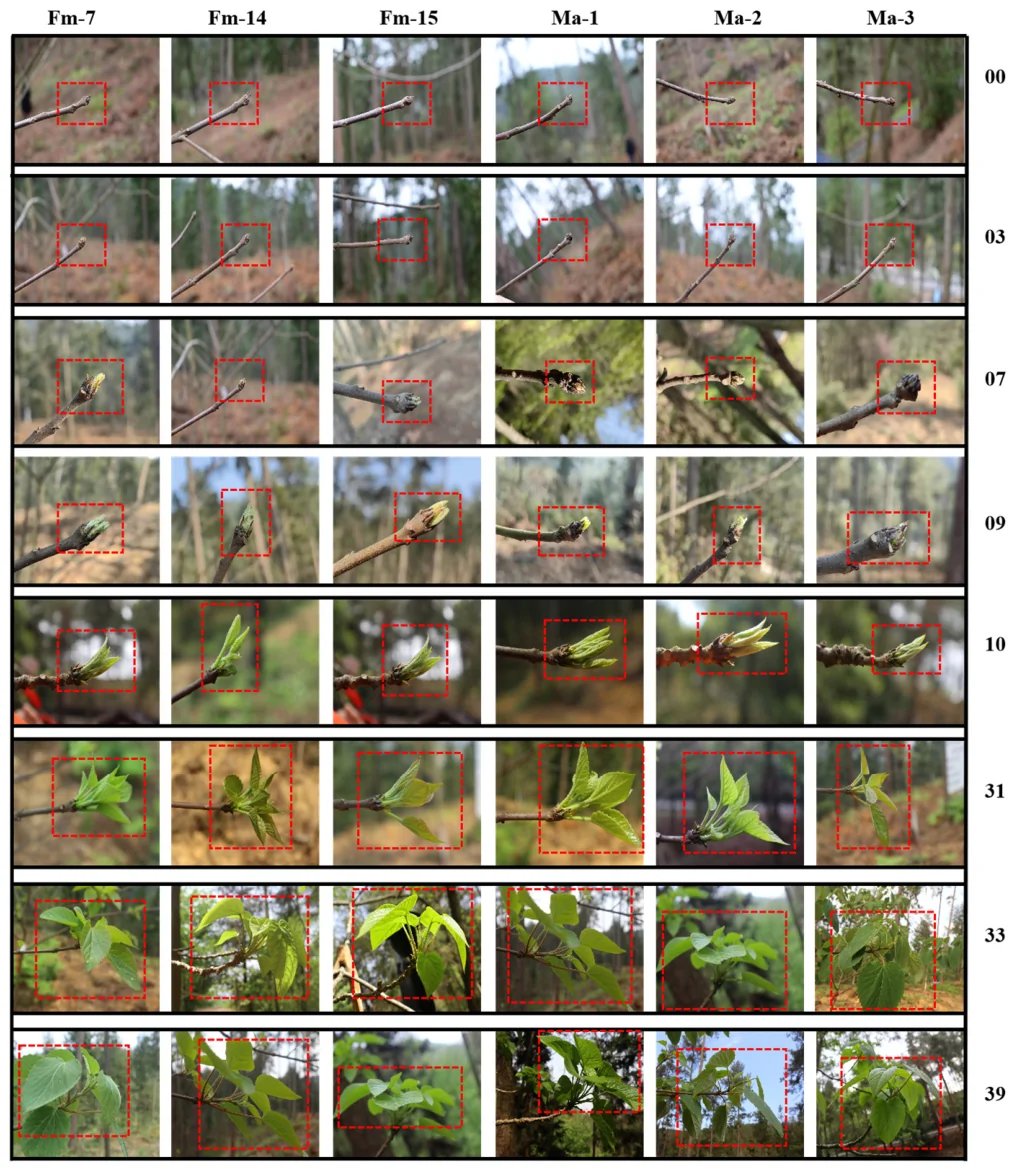
Vegetative growth stages of *I. polycarpa* according to the BBCH scale.

**Figure 3:**
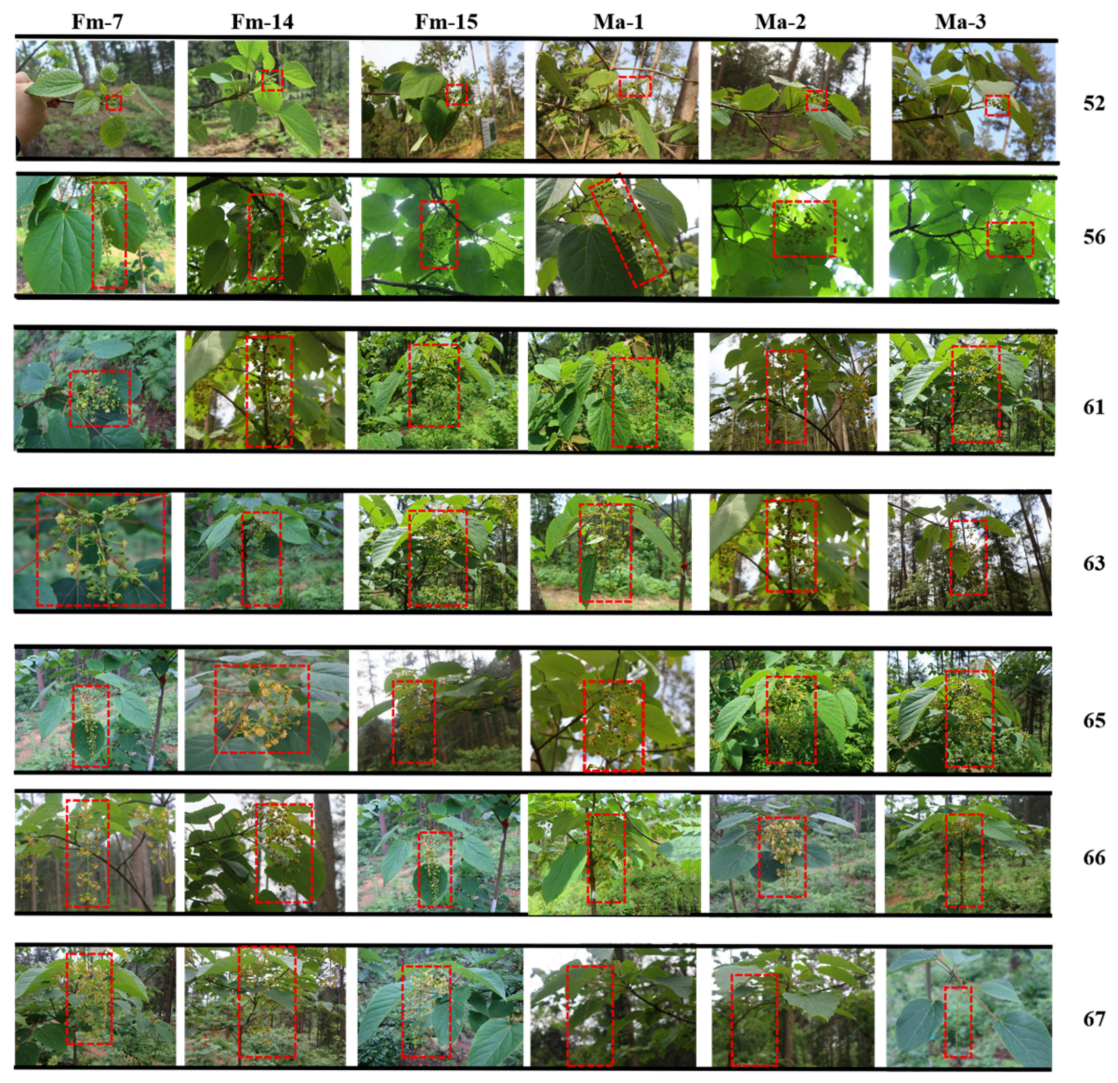
Reproductive growth stages of *I. polycarpa* (female and male) according to the BBCH scale.

**Figure 4:**
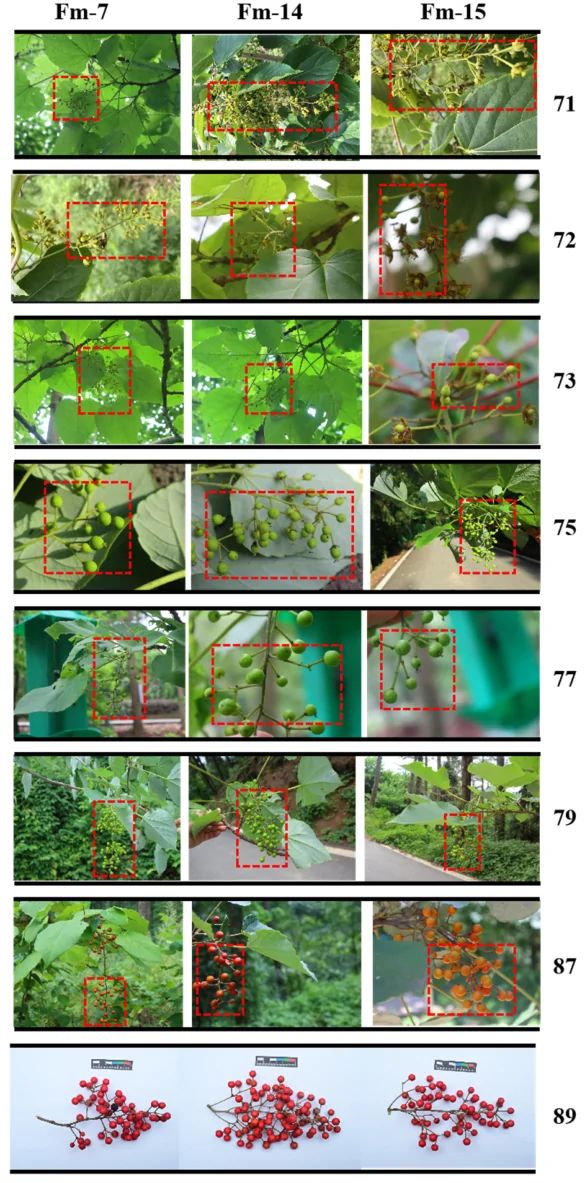
Reproductive growth stages of *I. polycarpa* (female) according to the BBCH scale.

**Figure 5:**
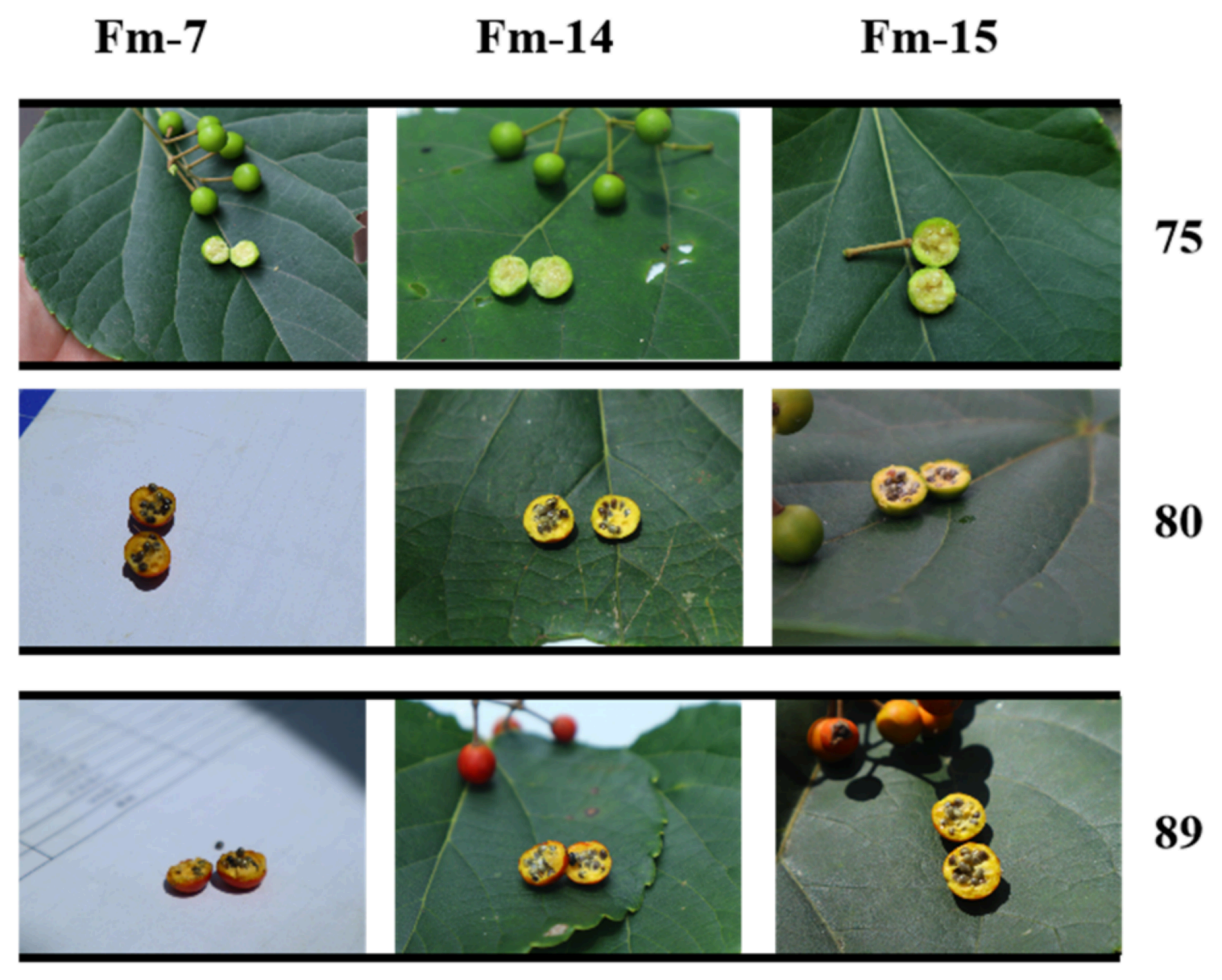
Seed growth stages of *I. polycarpa*.

**Figure 6:**
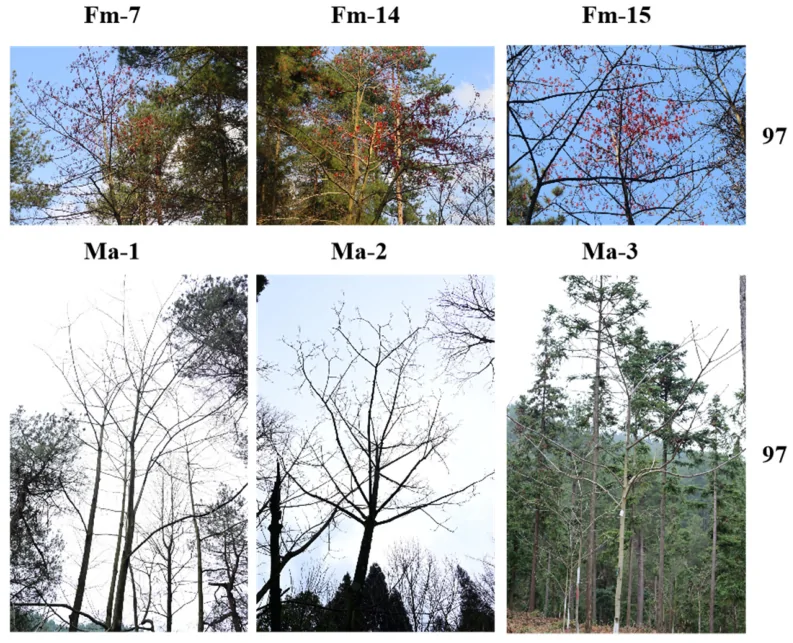
Dormant stages of *I. polycarpa* according to the BBCH scale.

**Figure 7:**
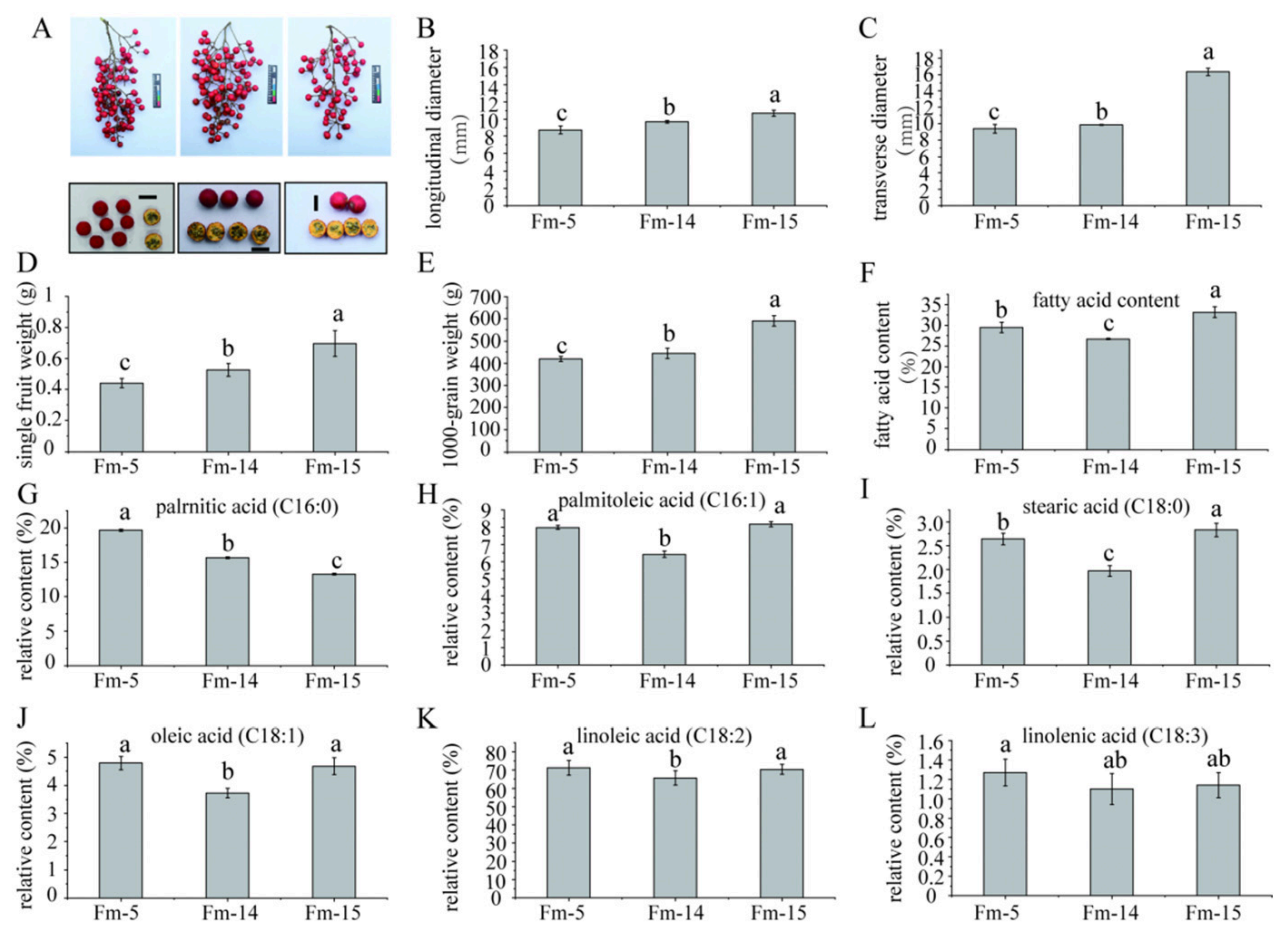
Fruit phenotypes, fatty acid content and composition of three *I. polycarpa* germplasm resources at BBCH stage 89. In A, the ruler = 6 cm, the black bar = 1 cm. (**A**) Mature fruits of three Idesia polycarpa germplasms; (**B**,**C**) Transverse diameter and longitudinal diameter of fruits; (**D**,**E**) Single fruit weight and 1000-seed weight of fruits; (**F**–**L**) The contents of fatty acid, Palrnitic acid (C16:0), palmitoleic acid (C16:1), stearic acid (C18:0), oleic acid (C18:1), linoleic acid (C18:2) and linolenic acid (C18:3). Different letters indicate significant differences (*p* < 0.05) within the different lines.

## Data Availability

Data will be made available upon request.
